# Emotional and Uncontrolled Eating as Mediators Between Perceived Stress and Adiposity Indicators in University Students: A Cross‐Sectional Study

**DOI:** 10.1002/brb3.71647

**Published:** 2026-08-11

**Authors:** Sahar Afeef, Sundus Malaikah, Nehal AlSharif, Noor Algoregry, Reoff Alobeidi, Eram Albajri

**Affiliations:** ^1^ Department of Clinical Nutrition, Faculty of Applied Medical Sciences King Abdulaziz University Jeddah Saudi Arabia; ^2^ Performance, Exercise, and Health Sciences Unit, King Fahad Medical Research Center King Abdulaziz University Jeddah Saudi Arabia

**Keywords:** body mass index, cognitive restraint, emotional eating, handgrip strength, mediation, perceived stress, Saudi Arabia, uncontrolled eating, university students, waist circumference

## Abstract

**Background:**

Perceived stress has been associated with maladaptive eating behaviors, yet evidence from young adults in non‑Western populations remains limited. This study examined whether eating behavior traits mediate the relationship between stress and adiposity indicators among university students.

**Methods:**

In a cross‐sectional study, undergraduate students (aged 18–26 years) completed Arabic versions of the Perceived Stress Scale (PSS‐10) and the Three‐Factor Eating Questionnaire‐Revised 18 (TFEQ‐R18) to assess perceived stress and three eating behavior traits (i.e., cognitive restraint, uncontrolled eating, and emotional eating). Anthropometric data, including body mass index (BMI), waist circumference (WC), and handgrip strength, were measured. Statistical analyses included group comparisons, Spearman correlations, and sex‐ and age‐adjusted parallel mediation to assess indirect effects of perceived stress on adiposity indicators via eating behaviors.

**Results:**

Of 297 students (66.7% female), males had higher BMI, WC, and handgrip strength (*p <* 0.001), while females reported greater perceived stress and cognitive restraint (*p ≤* 0.032). No direct association was found between stress and BMI (*b =* –0.014, 95% CI: –0.107–0.079) or WC (*b =* –0.037, 95% CI: –0.248–0.174). However, perceived stress was indirectly associated with higher BMI via emotional eating (*b =* 0.031, 95% CI: 0.004–0.066; β = 0.034) and higher WC via uncontrolled eating (*b =* 0.048, 95% CI: 0.002–0.118; β = 0.018), although these effects were very small. Cognitive restraint was not an indirect pathway for either outcome.

**Conclusion:**

Perceived stress showed very small indirect associations with BMI and WC via emotional and uncontrolled eating, respectively. These findings suggest potential behavioral correlates, but as cross‑sectional associations, they do not provide evidence of causal mediation or clinical significance and require longitudinal study.

## Graphical abstract

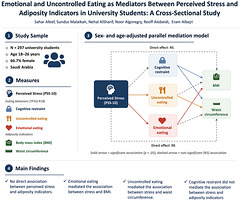

## 1 Introduction

Emerging adulthood (ages 18–25 years) is a critical period for determining lifelong health behaviors as it is more likely to involve substantial lifestyle changes, increased independence, and greater exposure to stressors (Arnett 2000; Nelson et al. 2008). This transition is accompanied by ongoing maturation of self‐regulatory capacities, greater independence in making food choices, and increased exposure to new academic, social, and environmental challenges (Arnett 2000; Nelson et al. 2008; Stok et al. 2018). As a result, health behaviors, and body composition during this period can have long‐term implications for the risk of obesity and related non‐communicable diseases (Nelson et al. 2008; VanKim et al. 2012). University students, in particular, navigate a unique combination of academic, social, and lifestyle transitions that may heighten vulnerability to maladaptive coping and weight gain (Assaf et al. 2019; Puente‐Hidalgo et al. 2024).

Perceived stress is a major determinant of health behaviors and outcomes (Schneiderman et al. 2005). It refers to the degree to which individuals view circumstances in their lives as unpredictable, uncontrollable, and overwhelming, indicating both exposure to stressors and individuals' perceived ability to manage them (Cohen and Williamson 1988). In a university setting, high perceived stress can stem from academic workload, exams, financial pressures, social challenges, sleep disruptions, and inconsistent eating patterns (Beiter et al. 2015; Stok et al. 2018; Strete et al. 2025). Stress has been associated with adiposity and cardiometabolic health indicators such as body mass index (BMI) and waist circumference, which are commonly used markers of weight status and central adiposity (Block et al. 2009).

However, the mechanisms underlying this association are complex, involving both physiological and behavioral pathways. Physiologically, stress activates the hypothalamic‐pituitary‐adrenal (HPA) axis, altering stress‐related hormones such as cortisol (McEwen 1998) and contributing to insulin resistance (Black 2006). These alterations may affect appetite regulation, food choices, reward sensitivity, eating control, fat distribution, and metabolic health (Adam and Epel 2007; Fardet and Fève 2014). Perceived stress may therefore help explain why certain students develop maladaptive eating habits, which can heighten their risk of weight gain and central adiposity.

Stress‐related eating varies among individuals. Stress has been associated with reduced energy intake in some individuals (Nagy et al. 2019), whereas others may increase intake, particularly of highly pleasurable foods (Adam and Epel 2007; Yau and Potenza 2014). This variability suggests that the link between perceived stress and adiposity may depend not only on individual eating behavior but also on social and cultural factors (Folkman 2013; Torres and Nowson 2007, 2007). Examining these pathways across diverse cultural settings is therefore important for understanding whether the stress–eating–adiposity relationship is generalizable across populations or shaped by local context.

Specific eating behavior traits have been identified as potential mediators of the relationship between stress and energy intake (Dakanalis et al. 2023) and between stress and adiposity (Yılmaz et al. 2024). One such trait is emotional eating, defined as the tendency to consume food in response to negative emotions (Dakanalis et al. 2023). From an emotional regulation perspective, emotions can influence eating in several ways, including altering appetite, increasing impulsive intake, or motivating food consumption as a short‐term coping strategy to reduce distress and regulate unpleasant emotional states (Macht 2008). Emotional eating may therefore serve as an affect regulation mechanism linking perceived stress to increased energy intake and weight gain (Dakanalis et al. 2023; Pacheco et al. 2021).

From a reward and cognitive control perspective, stress may also contribute to uncontrolled eating, characterized by disinhibited or unplanned eating and difficulty regulating food intake once eating has begun (De Lauzon et al. 2004). Mechanistically, it is suggested that chronic stress induces alterations in the mesolimbic dopaminergic system and stress‐motivation circuits, thereby increasing reward sensitivity, food preference, and motivation for hyperpalatable foods (Adam and Epel 2007; Yau and Potenza 2014). In parallel, stress may impair prefrontal‐cortex‐supported executive functions involved in eating regulation, including inhibitory control, attention regulation, and goal‐directed decision‐making (Arnsten 2009). Under stress, reduced top‐down control may make it more difficult to resist food cues, regulate portion size, or stop eating once eating has begun. In contrast, cognitive restraint reflects the conscious restriction of food intake to control body weight and may represent an intentional regulation pathway. Although cognitive restraint is associated with reduced energy intake (Stinson et al. 2019), however, it may increase the susceptibility to overeating and disordered eating patterns (Herman and Polivy 1980; Laessle et al. 1989a; Pacheco et al. 2021). Together, these mechanisms provide a theoretical basis for examining these eating behavior traits as a behavioral pathway linking perceived stress with adiposity.

Although previous research has linked perceived stress, emotional eating, uncontrolled eating, and adiposity, evidence remains limited among young adults in non‐Western university settings. In Saudi Arabia, perceived stress appears to be common among university students, with one study reporting that approximately 59% of university students report experiencing stress, with significantly higher prevalence among females (Mohamed et al. 2020). Despite this concerning prevalence, few Saudi studies have examined the link between stress and dietary patterns. Stress has been linked to unhealthy eating habits, such as increased consumption of sweets, fast food, and sugary drinks (Almogbel et al. 2019; Mohamed et al. 2020), and reduced adherence to healthy diets (Shatwan and Alzharani 2024), which may increase obesity risk. However, these studies have concentrated on dietary intake or general eating habits, offering limited insight into whether particular eating behavior traits statistically account for the link between perceived stress and measurable adiposity (Almogbel et al. 2019; Mohamed et al. 2020; Shatwan and Alzharani 2024).

To our knowledge, there is limited evidence on whether stress‐related eating behaviors are similarly or differently linked to overall and central adiposity among Saudi university students. This distinction matters because BMI indicates overall body size, while waist circumference specifically measures central adiposity. Analyzing both can help determine whether emotional eating, uncontrolled eating, and cognitive restraint have distinct associations with adiposity indicators. Therefore, this study aimed to examine the associations between perceived stress, eating behavior, and anthropometric indicators of adiposity and to assess whether eating behavior mediates the relationship between perceived stress and adiposity indicators in healthy Saudi University students.

## 2 Methods

### 2.1 Study Design and Participants

This cross‐sectional study was conducted among undergraduate students at King Abdulaziz University (KAU), Jeddah, Saudi Arabia, in February 2024. Eligible participants were apparently healthy male and female undergraduate students aged 18 to 26 years, enrolled full‐time in various academic disciplines at KAU. Exclusion criteria included the presence of self‐reported chronic diseases such as diabetes or hypertension, pregnancy or breastfeeding within the past 12 months, or any cognitive or physical condition that could impair participation in the study. Sample size was estimated using GPower 3.1, informed by a previous study (Yılmaz et al. 2024), which reported a moderate association between perceived stress and BMI. This calculation was based on detecting this association rather than on mediation‐specific requirements. Assuming a conservative effect size of f^2^ = 0.08, α = 0.05, and a power of 0.80, the required sample size to detect associations between perceived stress, eating behavior, and BMI was 268 participants. To account for potential exclusions and missing data (∼20%), a target of 322 participants was set. A total of 339 participants were recruited using a convenience sampling strategy, and after applying exclusion criteria, 297 participants were included in the final analysis. Ethical approval was obtained from the Biomedical Ethics Research Committee of King Abdulaziz University (Ref. No. 24‐439), and all participants provided informed consent before their inclusion in the study. All procedures were conducted in accordance with the ethical standards of the Declaration of Helsinki.

### 2.2 Data Collection

Data collection involved a combination of in‐person assessments and self‐administered questionnaires. Participants were recruited through convenience sampling via campus announcements, flyers, and classroom invitations. Those who expressed interest attended scheduled sessions at university laboratories, where trained researchers conducted anthropometric and handgrip strength measurements, as detailed below. Participation was entirely voluntary; no financial compensation, course credit, or other incentives were offered, and students were free to withdraw at any point without consequence. Following the physical assessments, participants completed an online questionnaire developed using Google Forms. The questionnaire comprised three sections: (1) Sociodemographic and Lifestyle Characteristics, (2) the 10‐item Perceived Stress Scale (PSS‐10), and (3) the eating behavior questionnaire.

### 2.3 Anthropometric and Muscular Strength Measurements

Anthropometric measurements were performed by BSc Clinical Nutrition students who had completed a formal nutrition assessment module, following the World Health Organization (WHO) anthropometric assessment protocol (World Health Organization 2011). Before data collection, all researchers attended a standardization session in which procedures were reviewed and practiced under the direct supervision of the principal investigator (SA). Participants were asked to remove footwear and heavy outer clothing before all measurements were taken. Height was measured to the nearest 0.1 cm using a stadiometer (Seca, Hamburg, Germany) and weight to the nearest 0.1 kg using a calibrated digital scale (Detecto SlimPRO, USA). Body mass index (BMI) was calculated as weight in kilograms divided by height in meters squared and categorized using WHO criteria: underweight (<18.5), normal weight (18.5–24.9), overweight (25.0–29.9), and obese (≥30.0) (World Health Organization 2011). Waist circumference (WC) was taken midway between the lower rib margin and the iliac crest with a non‐elastic measuring tape (World Health Organization 2011). Abdominal obesity was classified using sex‐ and BMI‐specific WC cutoffs recommended by the International Atherosclerosis Society (IAS) and the International Chair on Cardiometabolic Risk (ICCR) Consensus Statement (Ross et al. 2020). This approach accounts for increasing waist circumference cut‐offs across BMI categories to improve the identification of individuals at elevated cardiometabolic risk. Given the lack of validated Saudi‐specific cut‐offs, Jordanian thresholds were applied as the most geographically and culturally proximate available reference values (Ross et al. 2020). Muscular strength was assessed via maximum handgrip strength using a digital dynamometer (CAMRY EH101, China). Participants were seated with their elbows flexed at 90 degrees and instructed to squeeze the device with maximum force for 3–5 s. Three trials were conducted for each hand, with brief rest intervals between attempts. The highest value was recorded for analysis.

In the Sociodemographic and Lifestyle section, participants reported their age, sex, academic year, field of study (medical or non‐medical), marital status (married or unmarried), employment status (employed or unemployed/student), household income (<12,000 or ≥12,000 SAR), and living arrangement (living with parents/relatives, spouse, or roommate/friend). Lifestyle information included food‐preparation responsibility (self, others, or restaurant) and the frequency of eating out or ordering takeout.

In the Perceived Stress section, the validated Arabic version of the 10‐item Perceived Stress Scale (PSS‐10) was used to assess perceived stress (Chaaya et al. 2010; Cohen and Williamson 1988). This instrument assesses the degree to which situations in life are appraised as stressful over the past month. Items are scored on a 5‐point Likert scale, ranging from 0 (never) to 4 (very often). Positively worded items (Items 4, 5, 7, and 8) were reverse‐scored, and the total score was computed by summing all item responses, yielding a range from 0 to 40. Higher scores indicate greater perceived stress. Participants were classified into three stress categories based on established cutoffs: low (≤13), moderate (14–26), and high (≥27). The Arabic version of the PSS‐10 has previously demonstrated acceptable internal consistency (Cronbach's α = 0.74) (Chaaya et al. 2010), which is consistent with the present study's findings (Cronbach's α = 0.77).

In the Eating Behaviors section, the validated Arabic version of the Three‐Factor Eating Questionnaire‐Revised 18 (TFEQ‐R18) was used to assess three dimensions of eating behavior: cognitive restraint (CR), uncontrolled eating (UE), and emotional eating (EE) (Alhebshi et al. 2023; Karlsson et al. 2000). The TFEQ‐R18 consists of 18 items: items 1–17 are scored on a 4‐point scale, while item 18 is scored on an 8‐point scale, which was recoded into a 4‐point scale following the procedure described by de Lauzon et al. (2004) (De Lauzon et al. 2004). CR was assessed using six items (items 1, 5, 11, 12, 14, and 18); UE by nine items (items 2, 3, 4, 6–9, 13, and 15); and EE by three items (items 10, 16, and 17). Raw scores for each subscale were transformed to a 0–100 scale using the formula: [(raw score − lowest possible raw score) / possible score range] × 100 (De Lauzon et al. 2004). Higher scores reflect stronger tendencies toward the respective eating behaviors. The internal consistency of the Arabic TFEQ‐R18 in the present sample was acceptable, with Cronbach's alpha coefficients of 0.83 for UE, 0.82 for EE, and 0.64 for CR, comparable to those reported in the original Arabic validation study (α = 0.78, 0.78, and 0.59, respectively) (Alhebshi et al. 2023).

### 2.4 Statistical Analysis

Normality was assessed using the Shapiro–Wilk test; all continuous variables were non‐normally distributed except the PSS‐10 total score. Descriptive statistics are reported as mean ± SD for normally distributed data, median (IQR) for non‐normally distributed data, and frequencies (%) for categorical variables. For group comparisons, independent t‐tests were used for normally distributed continuous variables, Mann–Whitney U or Kruskal–Wallis tests were used for non‐normally distributed continuous variables, and chi‐square tests were used for categorical variables. When Kruskal–Wallis tests were significant, post hoc pairwise Mann–Whitney U tests were conducted using Bonferroni correction for three comparisons, with an adjusted significance threshold of *p <* 0.0167. Spearman's correlation coefficients assessed the associations among stress, eating behaviors, anthropometry, and handgrip strength because most variables were non‐normally distributed. Parallel mediation models were conducted using the PROCESS macro (Model 4), adjusting for sex and age. Path coefficients (a‐paths, b‐paths, direct, and total effects) are reported as unstandardized (b) with 95% CI and standardized coefficients (β). Indirect effects were estimated via bootstrapping (5,000 resamples) and reported as b with 95% CI and β; significance was inferred when the 95% CI excluded zero. The β values were interpreted against Cohen's (1988) benchmarks for standardized effects (small ≥ 0.10, medium ≥ 0.30, large ≥ 0.50) (Cohen 1988). All analyses were performed using SPSS version 29, with significance set at *p <* 0.05.

## 3 Results

### 3.1 Participant Characteristics

Sample characteristics (*n =* 297), overall and stratified by sex, are presented in Table 1. The median age was 20.0 years (IQR: 2.0), with males being older than females (21.0 (2.0) vs. 20.0 (2.0) years, *p <* 0.001). Males had significantly greater BMI (25.3 kg/m^2^ (9.05) vs. 20.6 kg/m^2^ (5.1)) and waist circumference (87.0 cm (20.0) vs. 64.0 cm (10.5)) compared to females (*all p <* 0.001). Based on BMI classifications, a larger proportion of males were overweight (24.2%) or obese (26.3%) compared to females (15.7% and 3.0%, respectively; *p <* 0.001). When sex‐specific BMI and waist circumference thresholds were applied, 11.2% of males were identified as at high cardiometabolic risk, compared with 2.0% of females (*p <* 0.001).

**TABLE 1:** Sample characteristics overall (*n =* 297) and stratified by sex.

Variable	Total (*n =* 297)	Male (*n =* 99)	Female (*n =* 198)	*p*‐value
**Age (years)**	20.00 (2.0)	21.0 (2.0)	20.0 (2.0)	**<0.001**
**Anthropometry**				
Height (cm)	161.0 (14.8)	173.8 (7.6)	157.0 (8)	**<0.001**
Weight (kg)	57.0 (24.7)	75.1 (27.1)	51.7 (13.7)	**<0.001**
BMI (kg/m^2^)	21.6 (6.9)	25.3 (9.05)	20.6 (5.1)	**<0.001**
Waist circumference (cm)	70.0 (21.5)	87.0 (20)	64.0 (10.5)	**<0.001**
**BMI category (kg/m^2^)**				**<0.001**
Underweight (< 18.5)	57 (17.8%)	8 (8.1%)	45 (22.7%)	
Normal weight (18.5 – 24.9)	157 (52.9%)	41 (41.4%)	116 (58.6%)	
Overweight (25 – 29.9)	55 (18.5%)	24 (24.2%)	31 (15.7%)	
Obese (≥ 30)	32 (10.8%)	26 (26.3%)	6 (3.0%)	
**Health risk based on sex‐specific cutoff based on BMI and WC**				**<0.001**
No risk	280 (94.9%)	87 (88.8%)	193 (98%)	
High risk	15 (5.1%)	11 (11.2%)	4 (2%)	
**Maximum handgrip strength (kg)**	23.0 (13.7)	36.6 (10.1)	19.7 (5.8)	**<0.001**
**Academic year**				**<0.001**
First (preparation) year	52.0 (17.5%)	3.0 (3.0%)	49.0 (24.7%)	
Second year	97.0 (32.7%)	36.0 (36.4%)	61.0 (30.1%)	
Third year	81.0 (27.3%)	26.0 (26.3%)	55.0 (27.8%)	
Fourth year	42.0 (14.1%)	22.0 (22.2%)	20.0 (10.1%)	
Fifth year	17.0 (5.7%)	10.0 (10.1%)	7.0 (3.5%)	
Sixth year	8.0 (2.7%)	2.0 (2.0%)	6.0 (3.0%)	
**Field of study**				**0.001**
Health and medical fields	103 (34.7%)	47 (47.5%)	56 (28.3%)	
Non‐medical fields	194 (65.3%)	52 (52.5%)	142 (71.7%)	
**Household income (SAR)**				0.054
< 12,000	200 (67.3%)	74 (74.7%)	126 (63.6%)	
≥ 12,000	97 (32.7%)	25 (25.3%)	72 (36.4%)	
**Living status**				**<0.001**
With parents or relatives	274.0 (92.3%)	81.0 (81.8%)	193.0 (97.5%)	
With friends or roommate	4.0 (1.3%)	2.0 (2.0%)	2.0 (1.0%)	
With spouse	2.0 (0.7%)	1.0 (1.0%)	1.0 (0.5%)	
Alone	17.0 (5.7%)	15.0 (15.2%)	2.0 (1.0%)	
**Employment status**				0.109
Student/unemployed	284.0 (95.6%)	92.0 (92.2%)	192.0 (97.0%)	
Employed	13 (4.4%)	7 (7.1%)	6 (3%)	
**Marital status**				0.253
Unmarried	291.0 (98.0%)	98.0 (99.0%)	193.0 (97.5%)	
Married	6.0 (2.0%)	1.0 (1.0%)	5.0 (2.5%)	
**Food preparation responsibility**				**0.033**
Self	60.0 (20.2%)	15.0 (15.2%)	45.0 (22.7%)	
Someone else	187.0 (63.0%)	60.0 (60.6%)	127.0 (64.1%)	
Restaurant	50.0 (16.8%)	24.0 (24.2%)	26.0 (13.1%)	
**Frequency of eating out or ordering takeout**				**0.002**
Rarely	39.0 (13.1%)	6.0 (6.1%)	33.0 (16.7%)	
Sometimes	121.0 (40.7%)	34.0 (34.3%)	87.0 (43.9%)	
Mostly	97.0 (32.7%)	38.0 (38.4%)	59.0 (29.8%)	
Always	40.0 (13.5%)	21.0 (21.2%)	19.0 (9.6%)	
**PSS‐10 total score**	19.6 ± 6.3	17.7 ± 5.7	20.6 ± 6.4	**<0.001**
**Perceived stress level**				**0.004**
Low	49.0 (16.5%)	22.0 (22.2%)	27.0 (13.6%)	
Moderate	203.0 (68.4%)	71.0 (71.7%)	132.0 (66.7%)	
High	45.0 (15.2%)	6.0 (6.1%)	39.0 (19.7%)	
**TFEQ‐R18 standardized scores**				
Cognitive restraint	50.0% (28.0%)	44.0% (23.0%)	50.0% (28.0%)	**0.032**
Uncontrolled eating	41.0% (30.0%)	41.0% (29.0%)	37.0% (31.0%)	0.077
Emotional eating	33.0% (45.0%)	33.0% (34.0%)	33.0% (45.0%)	0.182

*Note*: Values are presented as mean ± SD for normally distributed variables, median (IQR) for non‐normally distributed variables, and n (%) for categorical variables. *p*‐values refer to comparisons between male and female participants and were assessed using the chi‑square test, independent *t*‑test, or Mann–Whitney U test, as appropriate.

Abbreviations: BMI = body mass index; PSS‑10 = perceived stress scale‑10; TFEQ‑R18 = three‑factor eating questionnaire‑revised (18‑item).

Maximum handgrip strength was also significantly higher among males than females (36.6 kg (10.1) vs. 19.7 kg (5.8); *p <* 0.001). Statistically significant sex differences were observed in academic year, field of study, and living status (*p <* 0.001 for each). Males were more likely to rely on restaurants for meals (24.2% vs. 13.1%, *p* = 0.033) and to eat out more frequently (*p* = 0.002).

Mean total PSS‐10 score was significantly higher in females than males (20.6 ± 6.4 vs. 17.7 ± 5.7; *p <* 0.001), and a greater proportion of females were classified in the high‐stress category compared to males (19.7% vs. 6.1%; *p* = 0.004). Cognitive restraint scores were significantly higher in females compared to males (50.0% (28.0%) vs. 44.0% (23.0%); *p* = 0.032), whereas differences in uncontrolled eating and emotional eating scores between sexes were not statistically significant.

### 3.2 Correlations between Eating Behaviors, Stress, Anthropometry, and Muscular Strength

Spearman's rank correlation coefficients are presented in Table 2. Perceived stress was positively associated with emotional eating (ρ = 0.20, *p <* 0.01) and, to a lesser extent, with uncontrolled eating (ρ = 0.12, *p <* 0.05). Emotional eating was strongly correlated with uncontrolled eating (ρ = 0.63, *p <* 0.01). Cognitive restraint showed a significant positive correlation with BMI (ρ = 0.24, *p <* 0.01), while both uncontrolled eating and emotional eating were also positively correlated with BMI and waist circumference (*p <* 0.05 for all). Perceived stress was negatively correlated with waist circumference (ρ = –0.16, *p* < 0.01) and handgrip strength (ρ = –0.23, *p* < 0.01), but not with BMI (*p* > 0.05).

**TABLE 2:** Spearman's correlations between eating behavior subscales, perceived stress, anthropometric measurements, and muscular strength.

Variable	Cognitive restraint	Uncontrolled eating	Emotional eating	PSS‐10 total score	BMI	Waist circumference
Uncontrolled eating	−0.16**	—				
Emotional eating	0.00	0.63**	—			
PSS‐10 Total Score	0.01	0.12*	0.20**	—		
BMI (kg/m^2^)	0.24**	0.13*	0.21**	−0.08	—	
Waist circumference (cm)	0.06	0.19**	0.16**	−0.16**	0.80**	—
Maximum handgrip strength (kg)	−0.06	0.08	0.04	−0.23**	0.37**	0.60**

*ρ* = Spearman's rho. *p* < 0.05 (*), *p* < 0.01 (**).

Abbreviations: BMI, body mass index; PSS‐10, perceived stress scale‐10.

### 3.3 Differences in Anthropometric and Eating Behaviors Across Perceived Stress Levels

Differences in anthropometric and eating behavior measures across stress levels (low, moderate, high) are summarized in Table 3. Waist circumference differed significantly across stress categories (*p* = 0.041), with the high‐stress group having a lower median WC compared to the low‐stress group (66.0 cm vs. 72.3 cm; post hoc *p* = 0.009). Handgrip strength decreased significantly with increasing stress (*p* = 0.006), with post hoc differences between low vs. moderate and low vs. high groups.

**TABLE 3:** Differences in anthropometric measurements, muscular strength, and eating behaviors across perceived stress levels.

Variables	Low stress (*n =* 49)^1^	Moderate stress (*n =* 203)^2^	High stress (*n =* 45)^3^	*p*	differences
**BMI (kg/m^2^)**	21.78 (5.48)	21.92 (7.32)	20.45 (7.06)	0.653	NS
**Waist circumference (cm)**	72.3 (20.4)	70.0 (22.5)	66.0 (12.8)	**0.041**	1–3
**Maximum handgrip strength (kg)**	25.7 (16.3)	23.1 (14.3)	20.5 (8.8)	**0.006**	1–2, 1–3
**TFEQ‐R18 standardized scores**					
**Cognitive restraint (%)**	50.0 (33.0)	50.0 (28.0)	50.0 (34.0)	0.580	NS
**Uncontrolled eating (%)**	33.0 (29.0)	41.0 (26.0)	44.0 (35.0)	**0.049**	NS
**Emotional eating (%)**	22.0 (44.0)	33.0 (45.0)	44.0 (45.0)	**0.006**	1–3

*Note*: Values are median (IQR).

*p*‐values from Kruskal–Wallis H tests. Post hoc Mann–Whitney U tests between groups used Bonferroni correction (*p* < 0.05/3 ≈ 0.0167).

Perceived Stress Level Groups: 1 = Low (PSS‐10 ≤ 13), 2 = Moderate (14–26), 3 = High (≥ 27). questionnaire‑revised (18‑item).

Abbreviations: NS = Not Significant, TFEQ‐R18 = three‑factor eating.

Emotional eating scores increased significantly with stress levels (*p* = 0.006), and pairwise comparisons showed a significant difference between low and high‐stress groups (low: 22.0% (44.0%); high: 44.0% (45.0%); *p* = 0.001). Uncontrolled eating also showed a significant increase across stress groups (*p* = 0.049), but the post hoc differences were not statistically significant after correction. No significant differences were found for BMI (*p* = 0.653) or cognitive restraint (*p* = 0.580) across stress groups.

### 3.4 Mediation Analysis

**FIGURE 1:**
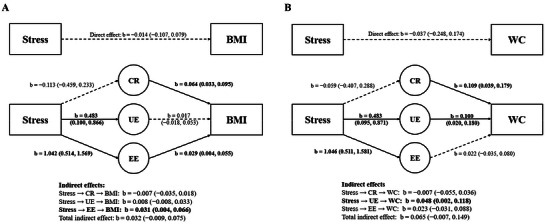
Path diagram of the parallel mediation model assessing the indirect effects of perceived stress on (A) BMI and (B) waist circumference via eating behaviors (cognitive restraint, uncontrolled eating, and emotional eating), adjusted for sex and age. Significant indirect effects were found via emotional eating for BMI and via uncontrolled eating for waist circumference (*p* < 0.05). Solid lines indicate significant paths; dashed lines are non‐significant.

Parallel mediation analyses adjusted for sex and age are presented in Table 4 and Figure 1. Perceived stress was positively associated with emotional eating (*b =* 1.042, 95% CI 0.514 to 1.569, *p* < 0.001) and uncontrolled eating (*b =* 0.483, 95% CI 0.100 to 0.866, *p* = 0.014), representing small‐to‐moderate effects (β = 0.226 and 0.146, respectively), but was not associated with cognitive restraint (*p* = 0.522). In the outcome equations (b‐paths), emotional eating (*b =* 0.029, 95% CI 0.004 to 0.055, *p* = 0.024) and cognitive restraint (*b =* 0.064, 95% CI 0.033 to 0.095, *p* < 0.001) were each independently associated with BMI, with small‐to‐moderate effects (β = 0.151 and 0.209, respectively). In contrast, uncontrolled eating (*b =* 0.100, 95% CI 0.020 to 0.180, *p* = 0.014) and cognitive restraint (*b =* 0.109, 95% CI 0.039 to 0.179, *p* = 0.002) were each independently associated with waist circumference, with small effects (β = 0.126 and 0.122, respectively).

**TABLE 4:** Path coefficients, direct, indirect, and total effects of perceived stress on BMI and waist circumference via eating behavior mediators, adjusted for sex and age.

	BMI (kg/m^2^), *N* = 297			Waist circumference (cm), *N* = 295		
	b (95% CI)	p	β	b (95% CI)	p	β
Regression path coefficients						
Perceived stress → eating behavior mediators						
Stress → CR (a_1_ )	−0.113 (−0.459, 0.233)	0.522	−0.038	−0.059 (−0.407, 0.288)	0.737	−0.020
Stress → UE (a_2_)	0.483 (0.100, 0.866)	**0.014**	0.146	0.483 (0.095, 0.871)	**0.015**	0.145
Stress → EE (a_3_)	1.042 (0.514, 1.569)	**<.001**	0.226	1.046 (0.511, 1.581)	**<0.001**	0.225
Eating behavior mediators → adiposity indicator						
CR → outcome (b_1_)	0.064 (0.033, 0.095)	**<.001**	0.209	0.109 (0.039, 0.179)	**0.002**	0.122
UE → outcome (b_2_)	0.017 (−0.018, 0.053)	0.343	0.063	0.100 (0.020, 0.180)	**0.014**	0.126
EE → outcome (b_3_)	0.029 (0.004, 0.055)	**0.024**	0.151	0.022 (−0.035, 0.080)	0.444	0.039
Direct effect (c′) and total effect (c)						
Direct effect (c′)	−0.014 (−0.107, 0.079)	0.762	−0.016	−0.037 (−0.248, 0.174)	0.732	−0.014
Total effect (c)	0.017 (−0.078, 0.112)	0.719	0.019	0.028 (−0.184, 0.241)	0.793	0.011
Indirect effects						
	b (95% CI)	p	β (95% CI)	b (95% CI)	p	β (95% CI)
Stress → CR → outcome	−0.007 (−0.035, 0.018)	—	−0.008 (−0.040, 0.020)	−0.007 (−0.055, 0.036)	—	−0.002 (−0.021, 0.013)
Stress → UE → outcome	0.008 (−0.008, 0.033)	—	0.009 (−0.009, 0.036)	**0.048 (0.002, 0.118)**	—	**0.018 (0.001, 0.045)**
Stress → EE → outcome	**0.031 (0.004, 0.066)**	—	**0.034 (0.004, 0.073)**	0.023 (−0.031, 0.088)	—	0.009 (−0.012, 0.033)
Total indirect effect	0.032 (−0.009, 0.075)	—	0.035 (−0.010, 0.082)	0.065 (−0.007, 0.149	—	0.025 (−0.003, 0.056)

*Note*: Two participants with missing waist circumference data were excluded from the waist circumference model (*N*  =  295).

Significant path coefficients (*p < *0.05) and significant indirect effects (95% CI excluding zero) are in **bold**.

Abbreviations: BMI  =  body mass index; b  =  unstandardized coefficient; CI  =  95% confidence interval; CR  =  cognitive restraint; UE  =  uncontrolled eating; EE  =  emotional eating; β  =  standardized coefficient.

Perceived stress showed no significant direct or total association with either BMI or waist circumference (all *p* ≥ 0.719). However, significant indirect effects through individual mediators were observed. Perceived stress was indirectly associated with higher BMI via emotional eating (*b =* 0.031, 95% CI 0.004 to 0.066) and with higher waist circumference via uncontrolled eating (*b =* 0.048, 95% CI 0.002 to 0.118), though these effects were very small (β = 0.034 and 0.018, respectively). A one‐standard‐deviation increment in perceived stress (i.e., 6.3 points in this sample) was associated with approximately 0.20 kg/m^2^ higher BMI via emotional eating and 0.30 cm higher waist circumference via uncontrolled eating.

## 4 Discussion

The main finding of this study was that perceived stress was not directly associated with BMI or waist circumference among Saudi university students. However, indirect associations were observed via emotional eating for BMI and uncontrolled eating for waist circumference. Cognitive restraint was positively associated with both adiposity indicators but did not statistically link perceived stress to either outcome. Females reported higher perceived stress and greater cognitive restraint, while males showed higher adiposity measures. Together, these findings suggest that maladaptive eating behaviors may be behaviorally correlated in the relationship between psychosocial stress and weight‐related outcomes (Adam and Epel 2007; Dakanalis et al. 2023; Torres and Nowson 2007).

Regional evidence supports the link between stress and eating behavior in Saudi college populations. A study based in Riyadh found that students reported overeating and sweet cravings under stress (Mohamed et al. 2020). Similarly, a sample of female students in Jeddah demonstrated that higher levels of emotional eating were correlated with snacking, take‐out consumption, and higher BMI (Mansoury 2024). In Taif, medical students under higher academic stress reported eating more frequently than usual (Aljaid et al. 2024). While these studies confirm associations between perceived stress and maladaptive eating behaviors, they have largely focused on dietary patterns and emotional responses, without examining whether specific constructs of eating behavior statistically link perceived stress to adiposity indicators in this population. Our findings extend this regional literature by showing that emotional and uncontrolled eating statistically link perceived stress to higher adiposity indicators in a Saudi university sample. These results are consistent with mediation patterns reported among young adults in other contexts, including European, Asian, and Latin American populations, where perceived stress was associated with greater emotional and uncontrolled eating and, higher adiposity (El Ansari et al. 2014; O'Connor et al. 2008; Pacheco et al. 2021; van Strien et al. 2020). Collectively, these findings point to emerging adulthood as a sensitive period during which psychosocial stress and maladaptive eating behaviors may converge in their association with adiposity (Nelson et al. 2008).

The absence of a direct association between perceived stress and adiposity indicators is theoretically consistent with models that position behavioral responses as potential links between stress and changes in body composition. Perceived stress may therefore be associated with greater emotional and uncontrolled eating tendencies, which are relevant to energy balance over time (Adam and Epel 2007; Yau and Potenza 2014). From a neurobehavioral perspective, these results align with affect‐regulation and reward‐control models of eating. Stress‐related emotional distress may trigger eating as a means of managing negative feelings, while increased reward sensitivity to palatable foods and diminished inhibitory control may contribute to overeating or difficulty stopping once eating has started (Adam and Epel 2007; Giddens et al. 2023; Macht 2008; McEwen 1998; Yau and Potenza 2014). This response may also be time‐dependent; acute stress can transiently suppress appetite through sympathetic activation, whereas prolonged or chronic stress promotes overeating via sustained HPA‐axis stimulation and cortisol elevation (Herman et al. 2016), a distinction relevant here, since the PSS‐10 captures stress over the preceding month rather than chronic, cumulative exposure. In this study, emotional and uncontrolled eating were the behaviors most consistently associated with perceived stress, with emotional eating appearing more relevant to overall adiposity and uncontrolled eating more relevant to central fat distribution. Although these indirect effects were statistically significant, they were very small and likely of limited clinical significance at the individual level. Accordingly, these findings should be interpreted cautiously as cross‐sectional associations rather than evidence of causal mediation or clinically meaningful effects. Nevertheless, because perceived stress is common among university students, including in Saudi Arabia (Fentahun et al. 2025; Mohamed et al. 2020), even small individual‐level associations may be relevant at a population level, identifying behavioral correlates worth examining in longitudinal studies.

In contrast to emotional and uncontrolled eating, cognitive restraint was positively associated with BMI and waist circumference in the sex‐ and age‐adjusted models but did not statistically link perceived stress to either adiposity indicator. This appears to reflect the absence of an association between perceived stress and cognitive restraint, even though cognitive restraint was independently related to both adiposity indicators. This pattern is consistent with classic restraint theory, which proposes that individuals with overweight or obesity report greater dietary restriction as a compensatory strategy in response to weight concerns rather than as a preventive behavior (Herman and Polivy 1980; Laessle et al. 1989b). Evidence from Western and Middle Eastern samples suggests that rigid forms of restraint may increase vulnerability to emotional and uncontrolled eating under stress (El Ansari et al. 2014; Pacheco et al. 2021). However, in the present study, cognitive restraint was not associated with emotional eating and was weakly but significantly negatively associated with uncontrolled eating, indicating that this pathway may be context‐dependent or influenced by population‐specific, cultural, or behavioral aspects such as concerns about weight, dieting history, food norms, or social expectations related to body weight. Thus, the present findings suggest that stress‐related adiposity pathways in this sample were more strongly linked to reactive eating behaviors than to deliberate dietary restraint.

The lack of a direct association between perceived stress and BMI in this sample mirrors findings from a large Japanese cross‐sectional study (Shimanoe et al. 2015) while contrasting with a population‐based study among US adults (Block et al. 2009). This discrepancy may partly reflect the temporal dynamics of stress measurement discussed above, alongside population‐specific differences in stress duration and metabolic exposure. In line with this, the indirect associations observed here suggest that behavioral correlates may be detectable in cross‐sectional data even where direct stress–adiposity associations are not (Adam and Epel 2007). The relatively young and apparently healthy nature of this sample may also reflect limited cumulative metabolic exposure and greater physiological adaptability in this age group, which may attenuate stress‐related adiposity within cross‐sectional designs (Nelson et al. 2008). Although BMI reflects overall body mass rather than fat distribution (Epel et al. 2000), perceived stress was not directly associated with waist circumference; the absence of a direct association is therefore unlikely to be explained by the limited sensitivity of BMI alone. Together, these findings suggest that stress‐related influences on adiposity indicators in this population may be associated with behavioral factors rather than with direct short‐term physiological mechanisms. Alternative explanations remain possible, such as higher adiposity may be associated with perceived stress or shared psychological and environmental factors affecting stress, eating behaviors, and adiposity simultaneously. Longitudinal research is needed to determine the directionality of these relationships.

Although sex differences were not a primary aim of this study, several anthropometric, behavioral, and contextual variables differed between male and female students. These differences are relevant, as sex‐related variation may be associated with body composition, perceived stress, eating behavior traits, and food‐related routines. In particular, the higher perceived stress and cognitive restraint observed among female students is consistent with prior research in university populations, where females reported significantly higher stress levels and more restraint‐related eating behaviors than their male counterparts (Bayram and Bilgel 2008; Mohamed et al. 2020). In addition, the significant differences in how males and females handle food preparation and how often they eat out or order takeout indicate potential variations in their food routines and exposure to external food environments. These contextual factors may be associated with eating patterns more broadly, through differences in meal structure, food accessibility, and reliance on convenience or restaurant foods (Lachat et al. 2012; Larson et al. 2006). Exploratory analyses, however, revealed that their associations with perceived stress were specific to female students and largely reflected the established sex differences in this sample, rather than independent contextual influences on eating behaviors. These variables are therefore retained as contextual descriptors, and their potential role warrants further investigation in future longitudinal studies with more balanced sex representation. Since detailed data on dietary intake, diet quality, and total energy consumption were not collected, these contextual factors should not be interpreted as direct causes of the observed mediation pathways. These sex differences may also reflect psychosocial and cultural determinants, particularly women's greater body‐image concern (Frederick et al. 2007), which aligns respectively with the higher cognitive restraint observed among female students in this sample.

These sex differences may help explain the inconsistent pattern observed between perceived stress and adiposity indicators. Perceived stress was negatively correlated with both waist circumference and handgrip strength. These two findings point in opposite directions of health implication, which suggests a shared sex confounder rather than a true stress effect. Female students reported higher stress, while male students showed higher waist circumference and handgrip strength. Handgrip strength was included only as a secondary indicator. It is also shaped by body size, muscle mass, activity, sleep, and sports participation, none of which were fully assessed. This finding should therefore be treated as exploratory, not as evidence that stress impairs physical function. In models adjusted for sex and age, perceived stress was not directly associated with BMI or waist circumference. The indirect association via uncontrolled eating was positive but very small. Together, these results point to a small, behavior‐specific association rather than a direct stress–adiposity relationship.

This study has several strengths. First, anthropometric assessments were obtained objectively by trained assessors, reducing the reporting and measurement bias inherent in self‐reported data. Second, perceived stress and eating behavior traits were assessed using validated Arabic versions of the PSS‐10 and TFEQ‐R18 in an understudied non‐Western university population. Finally, the study applied a pre‐specified, theory‐informed mediation model grounded in biobehavioral frameworks linking stress, eating behavior, and adiposity. This study also has several limitations. The cross‐sectional design precludes causal inference, and convenience sampling from a single institution, with a predominantly female sample, limits generalizability, as findings may reflect characteristics specific to this student population rather than broader patterns among young adults. The sex imbalance may also have influenced associations involving perceived stress, eating behaviors, and adiposity indicators; results should therefore be interpreted as preliminary and context‐specific. Self‐reported variables may be affected by recall or desirability bias, and because health status was based on self‐report rather than objective clinical assessment, some degree of misclassification cannot be excluded. Formal intra‐ and inter‐observer reliability indices were not assessed; however, assessors had relevant academic training in anthropometric measurement and participated in a supervised standardization session before data collection to reduce measurement error. The TFEQ‐R18 captures self‐reported eating behavior tendencies rather than actual dietary intake or energy consumption; higher emotional or uncontrolled eating scores should therefore not be interpreted as direct evidence of overeating or dietary excess. Physical activity, sleep quality, and objective dietary intake were not assessed and therefore could not be incorporated into the adjusted mediation models; residual confounding from these unmeasured lifestyle factors cannot be ruled out. Finally, the original sample size calculation targeted associations, not bootstrapped mediation. Simulation‐based benchmarks (Fritz and MacKinnon 2007) suggest *N =* 297 may be underpowered for the observed indirect effects (required *N =* 414–558), though these benchmarks do not account for the parallel mediation structure used. These findings, therefore, warrant replication in larger samples. Future studies should use longitudinal, multi‐university designs with more balanced sex representation, direct body composition assessment, and dietary intake measures to clarify the temporal and practical significance of the observed associations.

In summary, among Saudi university students, perceived stress was associated with maladaptive eating behaviors, particularly emotional and uncontrolled eating, which showed very small cross‐sectional indirect associations with higher BMI and waist circumference. These findings highlight behavioral correlates of stress‐adiposity indicators relationship worth further investigation and may help inform future longitudinal research on coping and eating behaviors during emerging adulthood.

## Author Contributions

**Eram Albajri**: Writing – original draft, Writing – review and editing, formal analysis. **Reoff Alobeidi**: writing – original draft, writing – review and editing, investigation. **Sundus Malaikah**: conceptualization, investigation, writing – original draft, methodology, writing – review and editing, supervision, resources. **Noor Algoregry**: writing – review and editing, writing – original draft, investigation. **Nehal Alsharif**: writing – original draft, writing – review and editing. **Sahar Afeef**: conceptualization, investigation, writing – original draft, methodology, writing – review and editing, formal analysis, supervision, resources.

## Funding

The authors have nothing to report.

## Ethics Statement

This study was approved by the Biomedical Ethics Research Committee of King Abdulaziz University (Ref. No. 24–439) and conducted in accordance with the ethical standards of the 1964 Declaration of Helsinki.

## Consent

Informed consent was obtained from all individual participants prior to their inclusion in the study.

## Conflicts of Interest

The authors declare no conflicts of interests.

## Data Availability

The data that support the findings of this study are available from the corresponding author upon reasonable request.
