# Correction to “Emotional and Uncontrolled Eating as Mediators Between Perceived Stress and Adiposity Indicators in University Students: A Cross‐Sectional Study”

**DOI:** 10.1002/brb3.71889

**Published:** 2026-10-28

Afeef, S., S. Malaikah, N. AlSharif, N. Algoregry, R. Alobeidi, and E. Albajri. 2026. “ Emotional and Uncontrolled Eating as Mediators Between Perceived Stress and Adiposity Indicators in University Students: A Cross‐Sectional Study.” *Brain and Behavior* 16, no. 8: e71647. https://doi.org/10.1002/brb3.71647

The Acknowledgments section was incorrect and should read as follows: “The authors acknowledge support from Prof. Hebah Kutbi and Dr. Wejdan Alghafari.”

The Funding section was missing and should read as follows: “This work was supported by the Indexed Publications Program (grant number: IPP 1397‐290–2025) through the Deanship of Scientific Research (DSR), King Abdulaziz University, Jeddah, Saudi Arabia. The supporter had no role in the design of the study; in the collection, analyses, or interpretation of data; in the writing of the manuscript; or in the decision to publish the results.”

We apologize for this error.

